# Multi-Modal Brain Tumor Detection Using Deep Neural Network and Multiclass SVM

**DOI:** 10.3390/medicina58081090

**Published:** 2022-08-12

**Authors:** Sarmad Maqsood, Robertas Damaševičius, Rytis Maskeliūnas

**Affiliations:** Faculty of Informatics, Kaunas University of Technology, LT-51386 Kaunas, Lithuania

**Keywords:** biomedical image processing, brain tumor, linear contrast stretching, deep learning, segmentation

## Abstract

*Background and Objectives:* Clinical diagnosis has become very significant in today’s health system. The most serious disease and the leading cause of mortality globally is brain cancer which is a key research topic in the field of medical imaging. The examination and prognosis of brain tumors can be improved by an early and precise diagnosis based on magnetic resonance imaging. For computer-aided diagnosis methods to assist radiologists in the proper detection of brain tumors, medical imagery must be detected, segmented, and classified. Manual brain tumor detection is a monotonous and error-prone procedure for radiologists; hence, it is very important to implement an automated method. As a result, the precise brain tumor detection and classification method is presented. *Materials and Methods:* The proposed method has five steps. In the first step, a linear contrast stretching is used to determine the edges in the source image. In the second step, a custom 17-layered deep neural network architecture is developed for the segmentation of brain tumors. In the third step, a modified MobileNetV2 architecture is used for feature extraction and is trained using transfer learning. In the fourth step, an entropy-based controlled method was used along with a multiclass support vector machine (M-SVM) for the best features selection. In the final step, M-SVM is used for brain tumor classification, which identifies the meningioma, glioma and pituitary images. *Results:* The proposed method was demonstrated on BraTS 2018 and Figshare datasets. Experimental study shows that the proposed brain tumor detection and classification method outperforms other methods both visually and quantitatively, obtaining an accuracy of 97.47% and 98.92%, respectively. Finally, we adopt the eXplainable Artificial Intelligence (XAI) method to explain the result. *Conclusions:* Our proposed approach for brain tumor detection and classification has outperformed prior methods. These findings demonstrate that the proposed approach obtained higher performance in terms of both visually and enhanced quantitative evaluation with improved accuracy.

## 1. Introduction

Among cancers, brain tumors now have the greatest preliminary cost per patient. The tremendous expansion in parts of cells in the brain can cause tumors in people of all ages. Brain tumors are produced by unconstrained enlargement of tissue in the brain or central spine that can interfere with normal brain function [1]. Based on the area, size, and position, these large tumor cells can be split into two types: cancerous (malignant) and non-cancerous (benign) cells [2]. The acute parts of cancer cells are known as primary and secondary tumor areas. The earliest stages of cancer cells are called benign and are declared as the primary tumor area. Primary brain tumors arise from brain cells and can be cured, their growth can be controlled by taking pertinent medications. Secondary (metastatic) brain tumors start in another part of the body and then spread to the brain. This tumor can only be cured, if the pretentious patient receives appropriate surgery or radiotherapy [3]. Because brain tumors harm the surrounding brain tissue, their progression should be closely monitored to ensure patient survival [4].

Meningiomas are tumors that invade the brain and spinal cord. The tumors are made of three layers of membranes known as meninges [5]. Meningiomas often present as off-axis lobar masses with well-defined edges [6]. Meningioma patients’ survival rates are determined by tumor size and location, and the patient’s age. Meningioma symptoms include clinging, headaches, and limb weakness. Most malignant meningiomas can be cured with early detection and effective treatment. Benign meningioma tumors are less than 2 mm in diameter while malignant meningioma tumors are up to 5 cm in diameter [7].

Magnetic Resonance Imaging (MRI) has become one of the most frequent procedures for detecting brain cancers, and many MRI methods can be utilized [8]. Accurate diagnosis and necessary treatment of patients is essential, as brain tumors can be hazardous, and the brain tumor disease at an early stage can be prevented only by complete brain area scanning to detect the tumor. Each MRI method has a varied composure time and can be utilized to detect different brain tissues [9]. On account of the uncertain structure and location of brain tumors, a single MRI modality is insufficient to detect irregularly shaped tumors in all brain regions. The MRI protocols of different sequences provide important contradictory information to identify tumor regions [10]. The application of different pulse sequences results in different types of MRIs, including: T1-weighted MRI that distinguishes tumor from healthy tissue, T2-weighted MRI outline areas of edema, resulting in clear image areas, T4-Gd MRI showed a bright signal at the tumor edge when contrast enhancement was used, and FLAIR MRI use water molecules to suppress signals to distinguish cerebrospinal fluid (CSF) from areas of edema.

Due to the structural complexity and variability of brain tumors, high volatility, and inherent properties of MRI data, i.e., variability of tumor size and shape, calculation of area, determination of uncertainty in segmentation area, and tumor segmentation are difficult tasks [11]. Some tumors, such as meningiomas, are simple to separate, but others, such as gliomas and glioblastomas, are more difficult [12]; hence, creating manual tumor segmentation is a tedious task, and in some instances, oncologists may observe changes in segmentation results due to differences in tumor appearance and shape. Therefore, it is imperative to present an automatic segmentation method to assist this strenuous task.

Manual recognition of brain tumors and tracking their progression is a time-consuming and error-prone operation [13]. We require an automated method to substitute the manual systems. Traditional methods involve labeling methods to detect diseased areas in the brain, and current methods cannot detect internal peripheral pixels, which are irreconcilable with brain tumor detection procedures. Owing to the area highlighted by the contrast agent and its clarity, we prefer MRI over Computed Tomography (CT). As a result, MRI modalities are used in numerous methods to detect brain cancers.

In recent years, many methods have been presented for the automatic classification of brain tumors, which can be divided into Machine Learning (ML) and Deep Learning (DL) methods based on feature fusion, feature selection and the learning mechanism. In ML methods, feature extraction and feature selection are fundamental to classification [14,15]. However, DL methods learn by extracting features directly from images. New DL methods, especially CNNs, offer excellent accuracy and are greatly employed in medical image analysis, including MRI analysis [16,17,18]. The disadvantages compared to traditional ML methods are also that it requires a large training dataset, high complexity of time, low accuracy for applications where small datasets are available and expensive GPUs which eventually elevate the user’s cost, although these disadvantages can be alleviated by using transfer learning [19]. In addition, choosing the accurate deep learning model can be an intimidating task, requiring knowledge of numerous parameters, training methods, and topologies. Numerous machine learning-based classifiers have been utilized for the brain tumor classification and detection, i.e., Support Vector Machine (SVM), Random Forest (RF), fuzzy C-mean (FCM), Convolutional Neural Network (CNN), Naïve Bayes (NB), K-Nearest Neighbor (KNN), Sequential Minimal Optimization (SMO), and Decision Tree (DT). The CNN implementation is very simple and requires less computational and spatial complexity. In general, these classifiers have received significant research attention due to the small dataset required for training, low computational complexity, and ease of adoption by unskilled individuals.

The following contributions in this work are proposed by the new brain tumor segmentation and classification method.

1. A linear contrast stretching method is used to improve the edge details of the original image as a pre-processing step;
2. Designed a custom 17-layered CNN architecture for brain tumor segmentation, which is trained from the scratch to recognize the tumor area;
3. We used transfer learning from modified MobileNetV2 to retrieve the selected datasets for the deep feature extraction;
4. To optimize feature selection, we use an entropy-based controlled method, where the best features are selected based on the entropy value. The final features are classified using a multi-class SVM classifier;
5. To confirm the stability of the proposed algorithm, a complete statistical analysis and comparison with the most modern methods are conducted.

The rest of the paper is organized as follows. The relevant study on brain tumor detection is described in Section 2. The proposed methodology is outlined in Section 3. The simulation setup and assessment matrices are specified in Section 4. Section 5 compares the performance of the proposed method with other current methods, and Section 6 gives a conclusion with future research aims.

## 2. Related Work

MR imaging is actively used in contemporary medical procedures to diagnose brain cancer [8,14]. This section thoroughly examines the reputation for excellence in the detection and classification of brain tumors.

In recent years, many researchers performed work on the detection, segmentation, and classification of brain tumors. The importance of this topic is pertinacious in the medical community [20,21,22]. This research work describes methods for the detection and segmentation of brain tumors. Methods to diagnose brain tumors include generative and discriminatory methods to distinguish brain images [17,23]. Maqsood et al. [4] demonstrated a brain tumor detection method based on fuzzy logic and the U-NET CNN architecture. Contrast enhancement, the fuzzy logic-based edge detection method, and U-NET CNN classification were used in this method. A contrast enhancement method is applied to the source images for pre-processing, followed by an edge detection method based on fuzzy logic to discover the edges in the contrast enhanced images, and finally a dual tree-complex wavelet transform is applied at various scale levels. The characteristics are generated from decomposed sub-band images, which are then classed using the U-NET CNN classification method, which distinguishes between meningioma and non-meningioma in brain imaging. The presented method was compared against various recently developed algorithms, and achieved an accuracy rate of 98.59%.

Sobhaninia et al. [24] used a LinkNet network with a CNN model for segmentation using brain MRI to train the model from different angles and perspectives to obtain good results and scores and achieved a dice score of 0.79. However, this network looks complex. Johnpeter et al. [25] detect and localize the tumors in brain MRI using an adaptive neuro-fuzzy inference classification method. This method used the histogram equalization method to enhance the tumor areas without using edge detection on the brain images. This work obtained an accuracy rate of 98.80%.

Togacar et al. [26] developed a BrainMRNet network using the modulo and hypercolumn method. First, the source images were pre-processed and afterwards they proceeded to the attention modulo. The attention modulo regulates the main areas of the image and directs the image to the convolutional layer. One of the primary strategies utilized in the convolutional layers of the BrainMRNet model is the hypercolumn. With this method, the attributes extracted from each layer are retained in the array tree of the last layer and attained an accuracy rate of 96.05%. Kibriya et al. [27] presented a feature fusion-based brain tumor classification method. The source images are pre-processed by minimum-maximum normalization method and then massive data extension is employed to pre-processed images to overwhelm the data problem. GoogLeNet and ResNet18 deep CNN models are used for the transfer learning and create a one feature vector and an SVM and KNN classifier is used for the final output and the obtained 97.7% accuracy. Sajjad et al. [28] developed a CNN based brain tumor detection and classification method. The authors used a Cascade CNN algorithm for the brain tumor segmentation and a fine tuned VGG19 is used for the tumor classification and attained an accuracy of 94.58%. Shanthakumar [29] used the MRI of the brain, using watershed segmentation to identify tumor regions. The segmentation method employs a series of predetermined labeling systems to maximize the accuracy of tumor segmentation and obtained an accuracy of 94.52%. Prastawa et al. [30] demonstrate how to segregate tumor areas in brain MRIs by detecting borderline pixels. This method detects only the aberrant borders of the tumor region however, not the inner border of the tumor region and hence achieved an accuracy of 88.17%.

Gumaei et al. [31] proposed a hybrid feature extraction method for brain tumor classification using a regularized extreme learning machine (RELM). The min–max normalization contrast enhancement method is used as a preprocessing step and the hybrid PCA-NGIST method is used for the feature extraction, and the RELM method is employed for the classification of the brain tumor. This work obtained an accuracy rate of 94.23%. Swati et al. [32] used a fine-tuned pre-trained VGG19 model on contrast-enhanced MRI (CE-MRI) to improve the results and obtained an average accuracy rate of 94.82%. Kumar et al. [33] proposed a brain tumor method using ResNet50 CNN model and global average pooling to resolve the problem of overfitting and obtained an average accuracy rate of 97.48%.

Although better results have been obtained with all of the above methods, however, there are still some shortcomings, i.e., many conventional methods use labeling methods to detect abnormal pixels in brain areas and current methods cannot diagnose the inside of the edge pixels, which is not suitable for many brain tumor detection algorithms [34,35]. Table 1 illustrates some of the current works with dataset information and results.

## 3. The Proposed Framework

**Figure 1:**
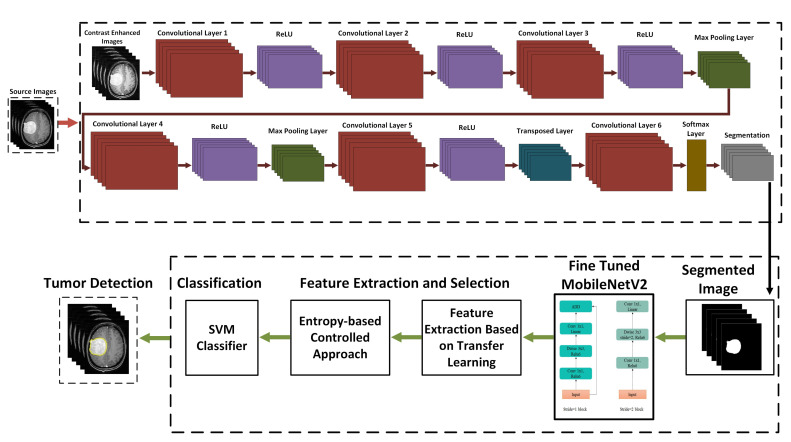
Proposed brain tumor segmentation and classification framework.

The proposed computer-aided design (CAD) method for the detection and classification of brain tumors includes contrast enhancement, image segmentation, feature extraction, feature selection and classification. The source brain images are first preprocessed using the linear contrast stretching method for the better visualization and the 17-layer CNN model is proposed for the tumor segmentation. A modified MobileNetV2 deep CNN model is used for the feature extraction, the entropy controlled method is employed for the feature selection and finally the M-SVM classifier is utilized for the brain tumor classification. Figure 1 shows a detailed scheme for the brain tumors’ segmentation and classification.

**Figure 2:**
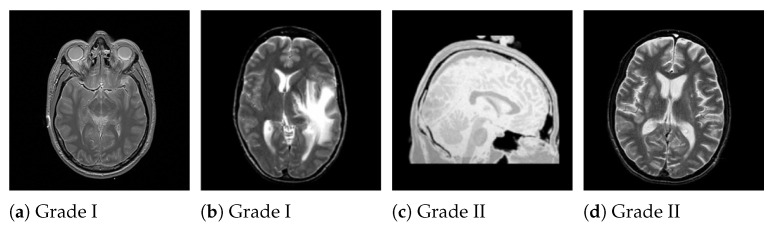
Examples of non-meningioma benign brain images.

**Figure 3:**
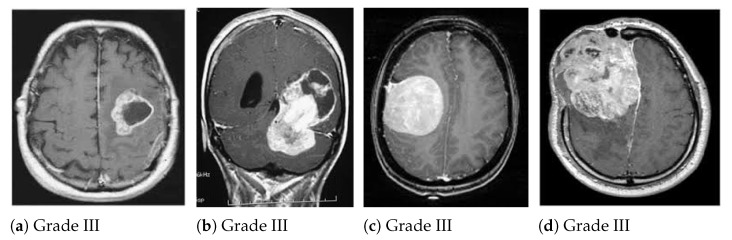
Examples of malignant meningioma brain images.

Figure 2 and Figure 3 illustrate the benign and malignant meningioma and benign and malignant non-meningioma brain images, respectively.

### 3.1. Contrast Enhancement

Contrast enhancement plays an important role and is the most effective method for refining the images that have low contrast [36]. Contrast stretching is performed in this task to enhance the visual contrast of tumors on MR imaging. Source MR imaging has several challenges, i.e., low contrast and similarity amongst healthy and diseased areas. Delineating the boundary between benign and malignant meningioma and non-meningioma complicates the detection process. Therefore, the linear contrast stretching is performed in order to refine the contrast while preserving the source MR image average brightness.

Let, r(x,y) denote the source image having size 256 × 256. The $i_{n}$, *n* = 0, 1, 2, 3, ..., *m*− 1 is the starting points of r(x,y) and $j_{n}$ and *n* = 0, 1, 2, 3, ..., *m*− 1 is the starting position of linear output enhanced image. The transformation function *m*− 1 is mathematically defined as follows:

(1) $$\zeta_{cs}\left(x,y\right)=\frac{j_{n}-j_{n-1}}{i_{n}-i_{n-1}}\times \left[x-i_{n-1}\right]+i_{n-1},$$

where $\zeta_{cs}\left(x,y\right)$ represents the linear stretched image. This image is further enhanced by function of contrast stretching, which is mathematically defined as follows:

(2) $$\zeta_{ce}\left(x,y\right)=\varphi \times \log\left[\zeta_{cs}\left(x,y\right)+r\right],$$

(3) $$\varphi \left(x\right)=\sum_{x,y=0}^{1}\zeta_{cs}\left(x,y\right),$$

where φ(x) represents the weighted value between 0 and 1.

**Figure 4:**
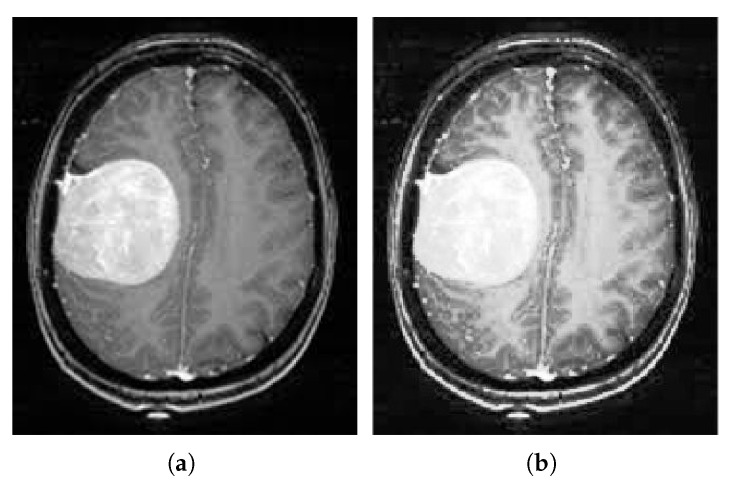
Linear contrast stretch outcomes. (**a**) Input brain MRI, (**b**) Final contrast stretch image.

Figure 4 shows the improvement of the brain MR image after employing linear contrast enhancement, the image gradients are well refined while preserving the information of the original image.

### 3.2. Tumor Segmentation

**Figure 5:**
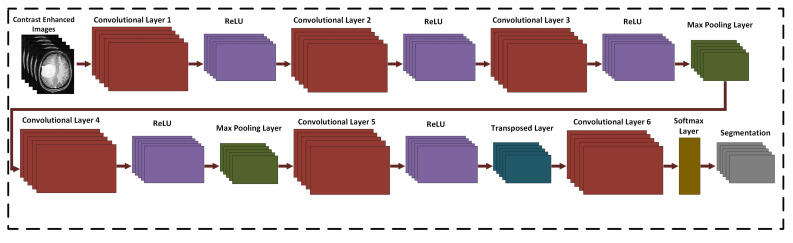
Proposed custom 17-layered CNN architecture for brain tumor segmentation.

The 17-layered CNN architecture (Figure 5) is proposed for brain tumor segmentation. This architecture consists of six layers of convolution, two layers of max-pooling, one transpose layer, five ReLU activation functions, a Softmax layer, and the pixel classification layer. The kernel size of the convolution layer is 3 × 3. The number of channels for the convolution layer are 32, 64, 128, 128, 256 and 2, respectively with stride of [1 1]. The enhanced image, with a dimension of 256 × 256 × 3 ,is fed to the network for tumor segmentation. More or fewer CNN layers have also been implemented for tumor detection but the proposed 17-layered CNN architecture is a unique model to accurately detect the tumor region in the brain MRI.

The first activation of the convolution layer is 256 × 256 × 32, the size of the weight matrix is 3 × 3 × 3 × 32, and the bias weight matrix with a dimension of 1 × 1 × 32. After the multi-pass modification, the convolution layer 2 weighting matrix is upgraded to the size of 3 × 3 × 32 × 64 and the upgraded bias matrix size is 1 × 1 × 64. A transposed convolution layer is used in layer number 14 with a 3 × 3 convolution function and 256 channels. The weight matrix size is 3 × 3 × 256 × 256 and the dimension of the bias matrix is 1 × 1 × 256. The output of the convolution layer is 256 × 256 × 2, which is transferred to the Softmax classifier. The layers are trained using the Adam optimizer with the mini-batch size of 128, the learning rate is 0.001 and the epochs number is 50.

The cross entropy function is then used to add a pixel label classification layer for the tumor segmentation. This function is mathematically stated as:

(4) $$\delta \left(\upsilon ,G\right)=-\frac{1}{W}\sum_{i=1}^{W}ln\left(C_{R}\right),$$

where υ denotes a size patch of 256 × 256 × 3, the true labels are denoted by *G*, the patch in the *i*-th image is denoted by *W*, and $C_{R}$ specifies the posterior probability of the real class *R*. Table 2 provides a detailed description of each layer used to train the neural network (NN).

Finally, we use morphological techniques to eliminate extraneous segments generated after segmentation or to improve the region of interest formed after segmentation. Opening, closure, erosion, and dilation are morphological processes. Morphological dilatation is used to enlarge the region of interest, whereas morphological erosion is used to eliminate the undesirable clusters created during segmentation. This frequently aids in the removal of undesirable picture areas following image segmentation, which is followed by an opening operation of erosion and dilation.

### 3.3. Modified MobileNetV2 for Feature Extraction

MobileNetV2 is a deep CNN framework designed for portable and resource-constrained situations. This model is based on an inverse residual structure, where the residual structure is linked to the bottleneck layer [37]. The motivation behind using the MobileNetV2 network has a reduced parameters number, is faster in performance, small size, and low-latency. MobileNetV2 has a total of 153 layers and the input layer size is 224 × 244 × 3.

As a unique solution to the inverse problem associated with representing brain tumors, we suggest a hybrid Long Short-Term Memory (LSTM) recurrent neural network integrated with reworked MobileNetV2 (as a base model), which is inspired by [38,39,40]. The hybrid model needs to estimate the system’s parameters when modeling different grades of tumor, taking into account tumor mass simulations generated by titrating the rates of proliferation, concentration-driven motility, and angiogenesis, as well as other factors associated with pathological and radiological features. The model needs to be capable of detecting changes in tumor model parameters. The reasoning is based on the fact that certain brain tumors differentiate to a higher, more malignant grade. This process is usually accompanied by an increase in the rates of proliferation, motility, or angiogenesis. The implementation of this goal is the early detection of a grade change and hence the output being a provision of the possible timely treatment action.

**Figure 6:**
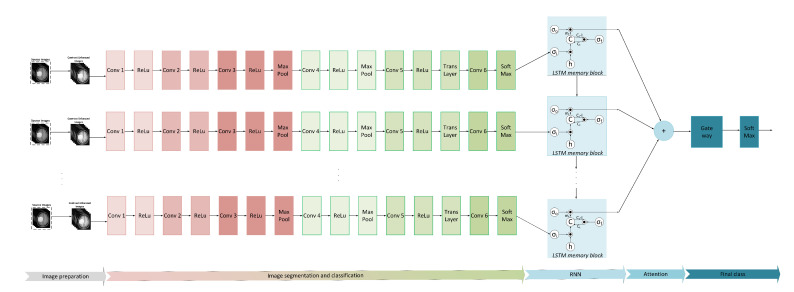
Structure of the MobileNetV2 and LSTM hybrid network ($\sigma_{o}$-output gate; $\sigma_{i}$-input gate; $\sigma_{f}$-forget gate).

First, we have modified the MobileNetV2 architecture with a completely new convolutional layer that includes benign and malignant meningioma and non-meningioma classes. These classes are called target labels. Then we use transfer learning (TL) to transfer the knowledge from the original network to the target network to acquire a new fitting CNN model. TL is used to train the fine-tuned network to extract features from the GAP layer for classification purposes, which are further used to help feed the LSTM (Figure 6). This element of the model can provide a labeled matrix with values for distinct picture areas and ridge lines, which aids in tumor detection. As a result, we take the complement of our image, apply RNN on the complemented image, and then negate the distance to discover the bright catchment basins that represent distinct areas.

As was mentioned, in contrast to other papers, where tumor segmenters were trained using only CNN versions, our technique used the impact of the LSTM memory cells to overcome the excessive vanishing error issue. One of the primary advantages of RNN modeling is that the LSTM can recall dependencies inside the sequence to establish the set of PDEs that the tumor is classified by, thereby improving system efficiency. The value of neuron in different layers and the mean centers initialized are fully dependent on categorizing the tumor using the RNN technology, the LSTM’s spatiotemporal parameters aid the model in recognizing concealed outlines in difficult frame-to-frame sequences.

Our hybrid technique divides images into dynamic zones. The RNN network creates the layers and neuron centroids. As a consequence, the picture from linear space is reconverted to the spatial domain, and the individual classification results are sub-displayed. For the majority of the brain images, the tumor is removed and is shown as one of the class findings based on the selected cluster.

### 3.4. Deep Feature Extraction Using Transfer Learning

A well-known deep learning method called transfer learning enables the use of a pre-trained model on a challenging research problem [22]. Utilizing TL has the significant benefit of requiring fewer input data while producing excellent results. It seeks to transfer knowledge from a source domain to a targeted domain, where the proposed problem with few labels is the targeted domain and the source domain is a pre-trained model with a large dataset. Typically, ImageNet, a sizable high-resolution image dataset, is used in the source domain [5]. There are 1000 image categories and more than 15 billion labels. The modified MobileNetV2 based CNN model is retrained using our datasets using transfer learning based feature extraction. TL is defined mathematically as follows:

The source domain $\zeta_{s}$ is defined as:

(5) $$\zeta_{s}=\left\{\left(m_{1}^{s},n_{1}^{s}\right),\ldots ,\left(m_{j}^{s},n_{j}^{s}\right),\ldots ,\left(m_{z}^{s},n_{z}^{s}\right)\right\}.$$

The learning tasks are $L_{s}$, $L_{\zeta }$, *$m_{x}^{s}$, $n_{x}^{s}$*∈ϕ.

The target domain $\zeta_{t}$ is defined as:

(6) $$\zeta_{t}=\left\{\left(m_{1}^{t},n_{1}^{t}\right),\ldots ,\left(m_{j}^{t},n_{j}^{t}\right),\ldots ,\left(m_{y}^{t},n_{y}^{t}\right)\right\}.$$

The learning tasks are $L_{t}$, *$m_{y}^{t}$, $n_{y}^{t}$*∈ϕ; (*x*,*y*) is the training size data, where *y*≪*x* and $n_{j}^{s}$ and $m_{j}^{t}$ are the labels for training data. The pre-trained model is trained on the target dataset according to this specification.

### 3.5. Feature Selection and Classification

Feature selection is a significant step in the applications area of deep learning [41]. Feature selection is utilized to enhance classification accuracy, remove redundancy amongst features and surpass only robust features for best classification. Here we used an entropy-based controlled method to choose the best features based on the entropy value. The method removes unnecessary and redundant attributes and selects just the highest priority features. Let F(x) be the feature vector of the texture along the *P* × *Q* dimensions, the entropy of the vector extracted F(x) is formulated as:

(7) $$F\left(x\right)=\sum_{s_{1}}^{x}\sum_{s_{2}}^{x}J\left(s_{1},s_{2}\right)\log J\left(s_{1},s_{2}\right),$$

(8) $$J\left(x\right)=-g\sum_{x=1}^{Q}v_{x}\ln v_{x},$$

where $s_{1}$ and $s_{2}$ represent the minimum existing and previous distance according to the selected features, $v_{x}$ represents the value of probability for each x-*th* feature, J(x) represents the computed entropy vector. A threshold function is used for the newly formed entropy vector, which returns only objects greater than the maximum probability feature $Hk_{D}$. The threshold function is computed as follows:

(9) $$\zeta_{s}\left(x\right)=\left\{\begin{array}{cc} X(x), & \mathrm{if}\;Hk_{D}\;\leq \;J(x)\\ 0, & \mathrm{Otherwise}. \end{array}\right.$$

Finally, the specified vector X(x) is forwarded to a multi-class SVM (M-SVM) classifier for the final classification where X(x)∈$\zeta_{s}\left(x\right)$. An approach for supervised machine learning method called SVM can be applied to classification issues. The data are transformed using a method known as the kernel trick, and based on these transformations, it determines the best output boundary. SVM works decently when there is a significant separation margin between categories, which is more efficient in high-dimensional spaces, and memory effective. The M-SVM classification identifies the benign and malignant meningioma and non-meningioma with the corresponding category.

## 4. Experimental Setup

This section discusses the simulation setup, dataset description of MR imaging, and evaluation measures.

### 4.1. Simulation Setup

The proposed method was implemented in the MATLAB R2021b on a laptop equipped with an Intel(R) Core(TM) i7-9750H processor, 16 GB RAM, an NVIDIA GTX 1650 GPU, and using the Microsoft Windows 11 environment.

### 4.2. Dataset

The experiment was assessed on brain MR images of meningiomas, gliomas, and pituitary. We assessed the proposed method on two publicly accessible datasets of brain tumor detection, i.e., figshare [42] and BraTS 2018 [43]. The brain figshare MRI dataset [42] contains entire 3064 T1-weighted contrast enhanced images of 233 patients including benign and malignant brain images. There is an MRI of the brain with meningioma containing a group of 708, an MRI of the brain with a glioma of a group containing 1426 images, and an MRI of the pituitary tumor of the brain group containing 930 images. These two datasets were utilized to compute the effectiveness of the proposed method for the detection, segmentation, and the classification of brain tumors. 

### 4.3. Evaluation Matrices

To evaluate the proposed method performance, Specificity (*Spe*), Sensitivity (*Sen*), Accuracy (*Acc*), and Dice coefficient index (*Dci*) metrics were used in this work. The highest of these stats indicates a superior performance. These metrics are described as follows:

(10) $$Acc=\frac{A_{TP}+A_{TN}}{A_{TP}+A_{TN}+A_{FN}+A_{FP}}\times 100\%$$

(11) $$Sen=\frac{A_{TP}}{A_{TP}+A_{FN}}\times 100\%$$

(12) $$Spe=\frac{A_{TN}}{A_{TN}+A_{FP}}\times 100\%$$

(13) $$Dci=\frac{2\times A_{TP}}{2\times A_{TP}+A_{FN}+A_{FP}}\times 100\%,$$

where $A_{FP}$ signifies the false positive, $A_{TP}$ signifies the true positive, $A_{FN}$ signifies the false negative, and $A_{TN}$ signifies the true negative.

## 5. Results and Discussion

### 5.1. Brain Tumor Segmentation Results

**Figure 7:**
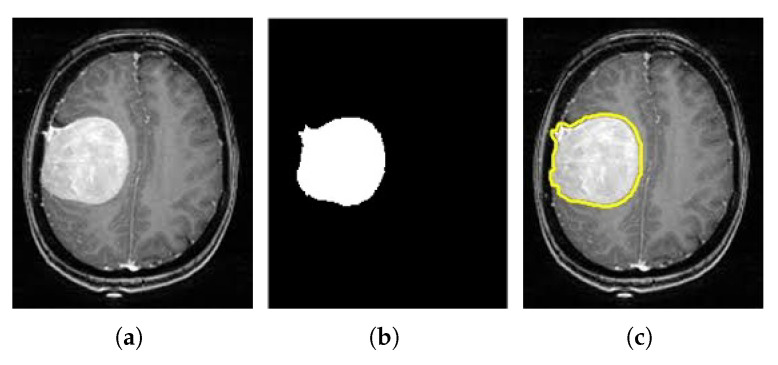
Meningioma brain image. (**a**) Source MRI, (**b**) Segmented tumor image, and (**c**) Extraction of tumor.

Segmentation has a significant role in medical imaging for preoperative and postoperative planning and early detection. Image segmentation divides an image into parts or areas based on the properties of the pixels in the image. In this work, the 17-layered CNN architecture is proposed for the tumor segmentation. Figure 7 displays the segmentation and tumor detection of the brain image.

### 5.2. Classification Results

Using two datasets—figshare and BraTS 2018—we report the classification results for the proposed M-SVM classifier. A 70:30 strategy was utilized to validate the proposed method and a 5-fold cross-validation was implemented.

#### 5.2.1. BraTS 2018 Dataset Results

As illustrated in Table 3, the simulation results of our approach attain the highest test detection accuracy of 95.41%. From Table 3, M-SVM stands out in Acc, Sen, Spe, and Dci by 97.47%, 97.22%, 97.94%, and 96.71%, respectively, using the BraTS 2018 dataset. It can be discerned from Table 4 that the proposed brain tumor detection method achieves a superior performance with M-SVM classification.

The proposed method is compared with other modern methods in terms of classification accuracy as shown in Table 4. All the methods were compared using the BraTS 2018 dataset and the performance was quantitatively assessed. All methods obtained a decent accuracy rate but were still unable to achieve the highest accuracy. Irfan et al. [44], Amin et al. [45], Narmatha et al. [46], and Khan et al. [47] have a classification accuracy rate of 92.50%, 93.85%, 92.50%, and 93.40%, respectively. Compared to all the existing methods the proposed method exhibits superior performance. The M-SVM classification method was utilized for the brain tumors detection and segmentation and attained a classification accuracy of 97.47%. From Table 4 we can conclude that the proposed method attains better classification accuracy than other methods.

#### 5.2.2. Figshare Dataset Results

As displayed in Table 5, the simulation results of our method achieve the highest test detection accuracy of 96.2%. From Table 5, M-SVM excels in Acc, Sen, Spe, and Dci by 98.92%, 98.82%, 99.02%, and 97.87%, respectively. It can be observed from Table 6 that the proposed brain tumor detection method achieves a superior performance with M-SVM classification.

**Figure 8:**
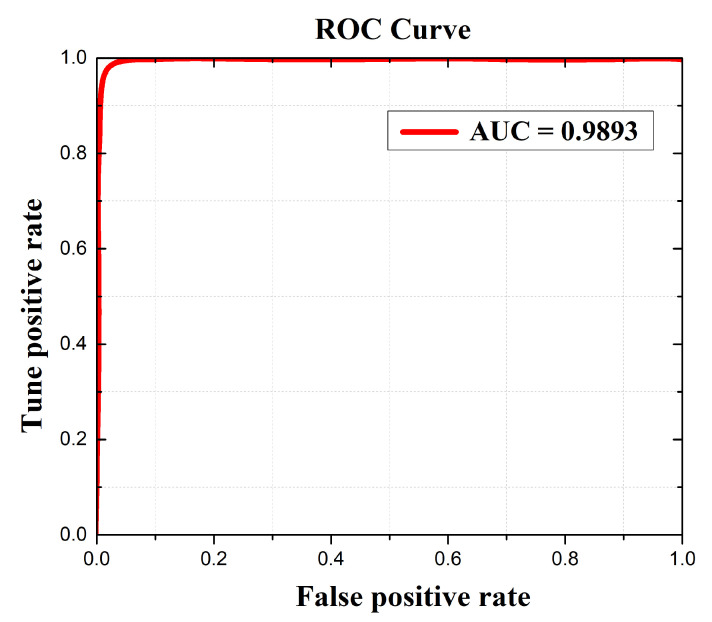
Receiver operating characteristic (ROC) curve of the proposed meningioma detection method.

**Figure 9:**
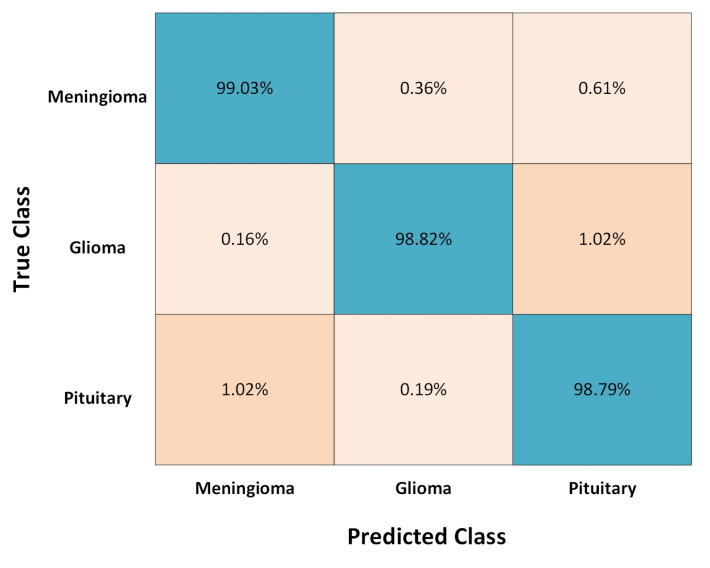
Confusion matrix for the classification of brain tumors.

The proposed method is compared with other modern methods in terms of classification accuracy as illustrated in Table 6. All the methods were compared using the figshare dataset and the performance was quantitatively assessed. All methods obtained a decent accuracy rate but were still unable to achieve the highest accuracy. Linear discriminant analysis (LDA) obtained a classification accuracy rate of 93.60%, while CNN and SVM achieved an accuracy rate of 96.50% and 94.63%, respectively. U-NET CNN and DarkNet-53 achieved better accuracy rates as compared to all the remaining methods with 98.59% and 98.59%, respectively, which is also very close to the proposed method. Noreen et al. [52], Anaraki et al. [54], Gumaei et al. [31], Sajjad et al. [28], and Swati et al. [32] all have almost the same classification accuracy rates of 94.34%, 94.20%, 94.23%, 94.58%, and 94.82%, respectively. Compared to all the existing methods the proposed method exhibits superior performance. The M-SVM classification method was utilized for brain tumor detection and segmentation, and 708 meningioma MRIs precisely classified 700 meningioma brain images with a classification accuracy of 98.92%. From Table 6 we can conclude that the proposed method attains better classification accuracy than other methods. The proposed method performance is also resolute using the Confusion Matrix and Receiver Operational Characteristics (ROC) curve. The Confusion Matrix and the ROC curve of the proposed method are illustrated in Figure 8 and Figure 9, respectively. The total execution time of the test is 15.64 s.

### 5.3. Explainability of the Results

**Figure 10:**
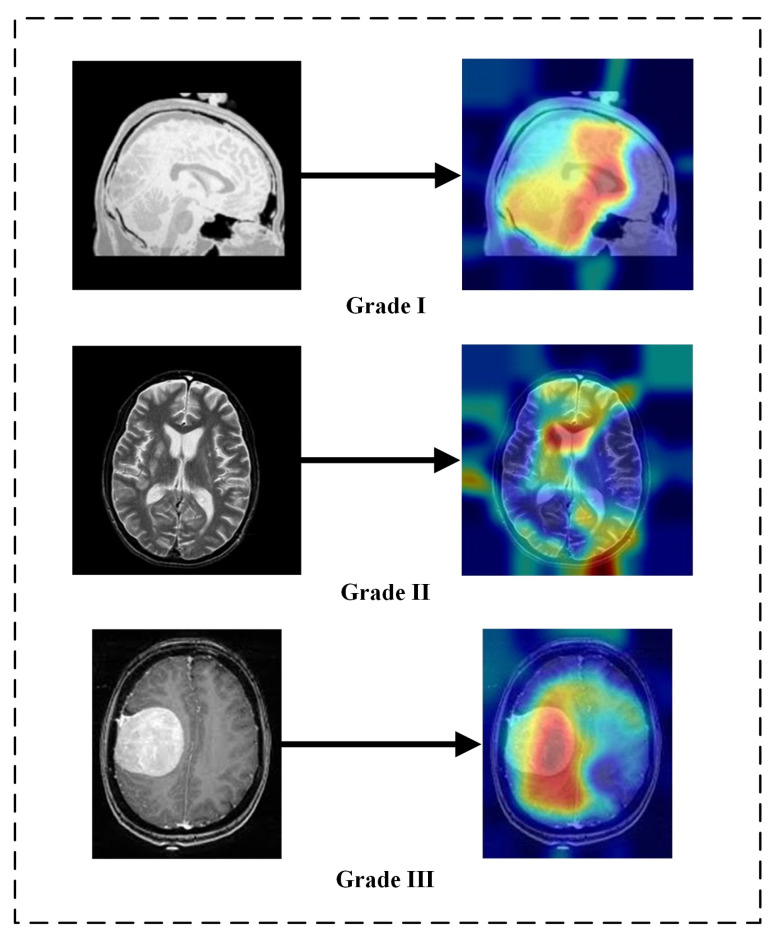
Localization of tumor using Grad-CAM on brain MRI.

The areas of an input image that contribute to the CNN final prediction can be visualized using Gradient-weighted Class Activation Mapping (Grad-CAM). Grad-CAM can provide a unique visualization for each class that is present in the image because it is class-specific. Grad-CAM creates a coarse localization map that highlights the key areas in the image for concept prediction by using the gradients of the target concept flowing into the final convolutional layer [56]. The localization of the tumor was performed using MR imaging classified into meningioma and non-meningioma tumor categories based on test data. Tumors were also located using Grad-CAM [56]. Red color represents the predicted tumor on the MR image and dark blue represents the background region of the MR image of the brain. Tumor localization imaging helps color map-based superpixel methods to enhance the localization of tumor pixels, which also leads to an increase in the dice index. Figure 10 shows localized tumor results for Grade I, Grade II, and Grade III.

### 5.4. Ablation Study

The ablation studies are performed to assess the influence of each component in the proposed methodology. The proposed method uses linear contrast stretching to refine the edges and a pre-trained MobileNetV2 is used for the feature extraction. For segmentation, the 17-layered CNN framework is developed to segment out the tumor region and the layers are trained using the Adam optimizer. The proposed system further explores the following research question: (1) How does the performance on the selected brain MRI dataset change when using different pre-trained CNN networks? (2) What effect does the optimizer have on the best pre-trained network’s performance? (3) How do changes to the cross-validation system affect MobileNetV2 classification performance? (4) How different multi-class classifiers affect MobileNetV2 classification performance on brain MRI datasets?

First, the performance of various pre-trained CNN architectures on the brain MRI dataset is evaluated. Table 7 briefly summarizes the performance comparison parameters and shows that MobileNetV2 outperforms other pre-trained deep learning networks.

The performance of the MobileNetV2 network is assessed using three optimization functions: (a) RMSprop; (b) stochastic gradient descent with momentum (sgdm); and (c) Adam. Table 8 illustrates that the MobileNetV2 network obtained the best performance with the Adam optimizer on the test dataset.

Different state-of-the-art deep learning networks are compared for the same brain tumor dataset using 5-fold cross-validation. The proposed method outperforms other methods in terms of accuracy as shown in Table 9.

The different multi-class classifiers (Fine tree, E-Bst tree, Fine KNN and M-SVM) are used for the selected dataset and Table 10 illustrates that MobileNetV2 obtained the best performance with M-SVM classifier with an accuracy of 98.92%, while its time performance is 15.64 s.

### 5.5. Limitations and Future Work

Our proposed model outperforms its competitors in terms of classification accuracy. The following advantages belong to our model as well.

- Because the suggested model employs a custom CNN, automatic feature extraction has been realized;
- Computational time is reduced because of the use of MobileNetV2;
- Because the Adam Optimizer is being used, the proposed method achieves quicker convergence;
- The entropy-based controlled feature selection scheme is employed to select the best features. Based on the entropy value, the entropy removes unnecessary and redundant attributes and selects only the highest priority features.

The major limitations of this study are as follows: (a) the methodology was implemented for 2-D MRI images; and (b) the feature selection method is slightly more time consumable. In the future, we will further use 3D brain imaging to achieve even more effective segmentation of brain tumors.

## 6. Conclusions

In recent years, demand for image-processing-based diagnostic computer systems has grown, enabling radiologists to speed up diagnosis while simultaneously assisting patients. The most deadly and life-threatening cancer, which affects many individuals globally, is the brain tumor. A variety of brain tumor segmentation and classification methods have been suggested to enhance medical image analysis. These algorithms, however, suffer from a number of drawbacks, including low contrast images, incorrect tumor region segmentation caused by some artifacts, a computationally complex method that needs more treatment time to correctly identify the tumor region, and existing deep learning methods need a large amount of training data to overcome overfitting.

The proposed brain tumor detection and classification scheme in this paper aims to address the aforementioned concerns. In our study, as a processing step, we used linear contrast stretching to refine detail at the edges of an image. The 17-layered CNN architecture is proposed for brain tumor segmentation and a modified MobileNetV2 architecture is used for feature extraction and trained using the transfer learning. Then, the features are selected using the entropy-based controlled method and the M-SVM framework is used to detect brain tumors.

An experimental study reveals that the proposed method obtained an enhanced performance in visual and comprehensive information extraction compared to current methods. The proposed classification method for the detection of brain tumors achieves an accuracy of 97.47% and 98.92%. The proposed method outperforms existing methods in terms of the detection and classification of brain tumors using MRI, as well as being more aesthetically pleasing and yielding superior results.

## Figures and Tables

**Table 1:** Detailed summaries of current research on the detection and classification of brain tumors.

References	Method and Methods Used	Modality	Results
Maqsood et al. [4]	Fuzzy logic and U-NET CNN classification	MRI	Accuracy = 98.59%
Sobhaninia et al. [24]	Linknet networks	MRI	Dice Score = 0.79
Johnpeter et al. [25]	Fusion based CANFIS classifier	MRI	Accuracy = 98.80%
Togacar et al. [26]	BrainMRNet	MRI	Accuracy = 96.05%
Kibriya et al. [27]	CNN, SVM, and KNN	MRI	Accuracy = 97.70%
Sajjad et al. [28]	Cascade CNN and VGG19	MRI	Accuracy = 94.58%
Shanthakumar [29]	Gray Level Co-occurrence and SVM	MRI	Accuracy = 94.52%
Prastawa et al. [30]	Geometric and Spatial Constraints	MRI	Accuracy = 88.17%
Gumaei et al. [31]	PCA-NGIST and RELM	MRI	Accuracy = 94.23%
Swati et al. [32]	Fine-tuned VGG19	MRI	Accuracy = 94.82%
Kumar et al. [33]	ResNet50 and Global Average Pooling	MRI	Accuracy = 97.48%

**Table 2:** Proposed CNN architecture layers.

Layers	Name	Type	Activations	Learnables
1	InputImage 256 × 256 × 3 images with “zero center” normalization	Input Image	256 × 256 × 3	-
2	Conv_1 32 3 × 3 × 3 convolution with stride [1 1] and padding ’same’	Convolution	256 × 256 × 32	Weights 3 × 3 × 3 × 32 Bias 1 × 1 × 32
3	ReLu_1 relu	ReLu	256 × 256 × 32	-
4	Conv_2 64 3 × 3 × 32 convolution with stride [1 1] and padding ’same’	Convolution	128 × 128 × 64	Weights 3 × 3 × 32 × 64 ias 1 × 1 × 64
5	ReLu_2 relu	ReLu	128 × 128 × 64	-
6	Conv_3 128 3 × 3 × 64 convolution with stride [1 1] and padding ’same’	Convolution	128 × 128 × 128	Weights 3 × 3 × 64 × 128 Bias 1 × 1 × 128
7	ReLu_3 relu	ReLu	128 × 128 × 128	-
8	Maxpool_1 5 × 5 max pooling with stride [1 1] and padding ’same’	Max Pooling	64 × 64 × 128	-
9	Conv_4 128 3 × 3 × 128 convolution with stride [1 1] and padding ’same’	Convolution	64 × 64 × 256	Weights 3 × 3 × 128 × 256 Bias 1 × 1 × 256
10	ReLu_4 relu	ReLu	64 × 64 × 256	-
11	Maxpool_2 5 × 5 max pooling with stride [1 1] and padding ’same’	Max Pooling	32 × 32 × 256	-
12	Conv_5 512 3 × 3 × 256 convolution with stride [1 1] and padding ’same’	Convolution	32 × 32 × 512	Weights 3 × 3 × 256 × 512 Bias 1 × 1 × 512
13	ReLu_5 relu	ReLu	32 × 32 × 512	-
14	Transposed conv 256 3 × 3 × 512 transposed convolution stride [1 1] and cropping ’same’	Transposed Convolution	32 × 32 × 512	Weights 3 × 3 × 256 × 512 Bias 1 × 1 × 512
15	Conv_6 1024 3 × 3 × 512 convolution with stride [1 1] and padding ’same’	Convolution	16 × 16 × 1024	Weights 3 × 3 × 256 × 1024 Bias 1 × 1 × 1024
16	Softmax	Softmax	1 × 1 × 256	-
17	Pixel class Cross entropy loss	Pixel Classification	-	-

**Table 3:** Quantitative assessment of the proposed method using M-SVM classification.

Proposed Method	
**Evaluation Metrics**	**Performance**
Accuracy (Acc)	97.47%
Sensitivity (Sen)	97.22%
Specificity (Spe)	97.94%
Dice coefficient index (Dci)	96.71%

**Table 4:** Performance comparison with existing methods.

Authors	Methods	Accuracy of Classification
Irfan et al. [44]	CNN, LBP, & PSO	92.50%
Amin et al. [45]	LSTM	93.85%
Narmatha et al. [46]	Brain-storm optimization	92.50%
Khan et al. [47]	DCT, CNN, & ELM	93.40%
Proposed Method	17-layered CNN, MobileNetV2 & M-SVM	**97.47%**

**Table 5:** Quantitative assessment of the proposed method using M-SVM classification.

Proposed Method	
**Evaluation Metrics**	**Performance**
Accuracy (Acc)	98.92%
Sensitivity (Sen)	98.82%
Specificity (Spe)	99.02%
Dice coefficient index (Dci)	97.87%

**Table 6:** Performance comparison with existing methods.

Authors	Methods	Accuracy of Classification
Maqsood et al. [4]	U-NET CNN	98.59%
Sajjad et al. [28]	VGG19 & image augmentation	94.58%
Gumaei et al. [31]	Regularized Extreme Learning MAchine	94.23%
Swati et al. [32]	Fine-tuned VGG19	94.82%
Kumar et al. [33]	ResNet50 & Global Average Pooling	97.48%
Cheng et al. [48]	Linear discriminant analysis (LDA)	93.60%
Badza et al. [49]	CNN	96.50%
Tripathi et al. [50]	SVM	94.63%
Ahuja et al. [51]	DarkNet-53	98.15%
Noreen et al. [52]	InceptionV3 & ensemble of KNN, SVM & RF	94.34%
Bodapati et al. [53]	Two channel DNN	97.23%
Anaraki et al. [54]	CNN & Genetic Algorithm	94.20%
Deepak et al. [55]	GoogleNet	97.10%
Proposed Method	17-layered CNN, MobileNetV2 & M-SVM	**98.92%**

**Table 7:** Performance comparison of various pre-trained models on brain MRI dataset.

Network	Images Size	Number of Parameters (in Millions)	Depth	Updated Layers	Training Accuracy
ResNet18	224 × 224 × 3	12	18	71	90.3%
DenseNet201	224 × 224 × 3	20	201	708	91.5%
SqueezeNet	227 × 227 × 3	2	18	68	92.7%
Inceptionv3	299 × 299 × 3	24	48	315	95.3%
DarkNet19	256 × 256 × 3	21	19	64	97.7%
MobileNetV2	224 × 224 × 3	4	53	154	98.8%

**Table 8:** Optimizer function’s performance with fine-tuned MobileNetV2 model on brain MRI dataset.

Optimizer	Accuracy	Sensitivity	Specificity
Sgdm	98.16%	97.71%	98.25%
RMSprop	98.78%	97.89%	98.86%
Adam	99.31%	98.76%	99.42%

**Table 9:** Accuracy comparison using different cross-validation system for brain MRI dataset.

Method	Cross-Validation	Accuracy
GoogleNet (Deepak et al. [55])	5-fold	97.10%
DarkNet-53 (Ahuja et al. [51])	5-fold	98.15%
U-Net CNN (Maqsood et al. [4])	5-fold	98.59%
Proposed	5-fold	98.92%

**Table 10:** MobileNetv2 based classification results for brain MRI dataset.

Method	Sensitivity	Accuracy	Time (s)
Fine tree	89.00%	89.20%	28.60
E-Bst tree	96.25%	96.40%	577.68
Fine KNN	97.50%	97.70%	37.78
M-SVM	98.82%	98.92%	15.64

## Data Availability

The data will be available upon request.
